# Training Miss Rosie: A nurse educator's pandemic journey to a new purpose

**DOI:** 10.1097/01.NURSE.0000918544.79454.31

**Published:** 2023-02-23

**Authors:** Park S. Balevre

**Affiliations:** At Chamberlain University, **Park Balevre** is an associate professor in the College of Nursing's Doctor of Nursing Practice program.

Much has been written about the mental health consequences of COVID-19 on nurses and healthcare workers.^1^-^3^ Some mitigators of mental health symptoms in nurses stemming from the pandemic include receiving a COVID-19 vaccine and using resilience and coping techniques.^4^,^5^ However, stress and burnout still take a tremendous toll on nurses and nursing faculty.^6^-^8^

During the pandemic, my wife, Sonia, and I witnessed nurses losing their personal and professional happiness—their hope and passion for life. This loss of hope suggests nurses need to rekindle that spark and find connection, purpose, balance, and renewal. Without these, professional and personal happiness disappears.

Sonia, a nursing professor, also experienced a loss of personal and professional happiness. Then, she decided to adopt a Royal Giant Schnauzer, Miss Rosie, and embark on a journey of becoming a dog trainer.

**Figure:**
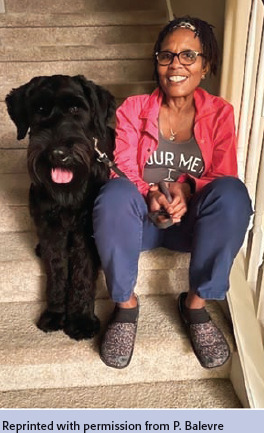
No caption available.

**Figure:**
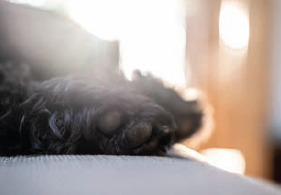
No caption available.

## Starting a life with Miss Rosie

Various studies have examined the psychodynamic benefits of dog ownership during the pandemic and the therapeutic benefits of animal-assisted therapy, companion animals, and pet therapy.^9^-^13^ The results of these studies suggest that pet ownership alters pandemic-generated negative attitudes, substitutes for diminished social support, reduces anxiety and stress, and improves emotional functioning and quality of life. During her journey, Sonia realized she had a new excitement for teaching because of Miss Rosie.

The first step was to locate a suitable trainer to guide her through the process. Sonia's son, who is a dog trainer, recommended someone near her area.

As we drove to pick up the puppy, Sonia made plans with excitement and anticipation—an animation that was new since the pandemic began. That exhilaration grew when we were introduced to our new 8-week-old family addition.

Miss Rosie would grow large, so daily discipline and a training regimen were necessary. We both excitedly anticipated this life change and felt like this commitment had renewed our spirits after the pandemic's isolating and restricting grasp.

As a full-time professor, Sonia's passion was teaching prelicensure baccalaureate nursing students. She especially enjoyed the clinical setting. She looked forward to each experience until the pandemic restricted and prevented hospital clinical visits. The resulting computer-based substitute robbed her of personal student contact and diminished her passion. However, Miss Rosie renewed her zest from the beginning. Miss Rosie reminded us that life was worthwhile.

## Bonding and connection

During the first year of the pandemic, Sonia often commented that she had to interact with students from across the room and use special care when communicating through a muffled mask. During this time, patients were separated from loved ones, the simple ungloved touch of someone who cared, and even their own nurses who were in full personal protective equipment. Nursing students and instructors were emotionally spent by these experiences and returned home disheartened and often questioned their profession choice.

For Sonia, Miss Rosie palliated those feelings and provided the expectation that there was a connection waiting at the end of the clinical day. She realized the importance of conveying this care and connection to her students and colleagues—and her enthusiasm became infectious.

## Finding a purpose

Akin to commitment is one's purpose in life; an overpowering purpose can vanquish COVID-19 burnout. I witnessed a comparable purpose in Sonia when she returned from a grueling clinical to greet Miss Rosie and me, eager to start the evening of training instead of surrendering to exhaustion. This new purpose helped her to focus her day and rekindle her joy in teaching.

## Work-life balance

Nursing faculty have historically reported work-life imbalance, and this disparity became greater during the COVID-19 pandemic.^14^ Sonia, too, had experienced such difficulty. After completing a 12-hour clinical rotation, she had to come home, shed her clinical uniform, demask, shower, and decompress. However, her work was not finished. Next, she would go online, enter attendance, and update clinical evaluations and reports. This often totaled a 20-hour workday. Exercise, relaxation, and nutrition were all affected. Her perception of a poor work-life balance created stress that prevented her from engaging in healthy lifestyle practices. Miss Rosie forced us to take control of our work-life balance to take care of her, train her, and grow with her.

## Renewal

Stress is not managed by reducing stressors but by detecting them and engaging in renewal activities.^6^ Miss Rosie lifted the veil to renewal activities hidden in plain sight.^6^ In my experience, renewal is a reset of attitude, creating a positive psycho-physiologic mindset. When the world is viewed through that lens, its colors are vibrant, and its possibilities are endless. For example, who knew a Giant Schnauzer could sit on the stairs? Such realizations bring renewal through a smile.

**Figure:**
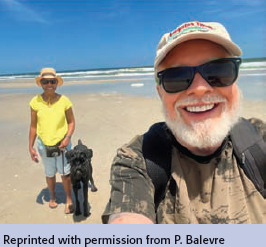
No caption available.

I invite you to renew your thinking and sit on the stairs with Sonia and Miss Rosie after a long day at work. Find your Miss Rosie—whether it is a Royal Giant Schnauzer, a person, a passion, or an aspect of your nursing role—that will rekindle your commitment, connection, and purpose, and, like Sonia, emerge from your pandemic journey with renewed hope and passion for what is to come.
